# Orientation-Invariant Spatio-Temporal Gait Analysis Using Foot-Worn Inertial Sensors

**DOI:** 10.3390/s21113940

**Published:** 2021-06-07

**Authors:** Vânia Guimarães, Inês Sousa, Miguel Velhote Correia

**Affiliations:** 1 Fraunhofer Portugal AICOS, 4200-135 Porto, Portugal; ines.sousa@fraunhofer.pt; 2 Faculty of Engineering, University of Porto, 4200-465 Porto, Portugal; mcorreia@fe.up.pt; 3 Institute for Systems and Computer Engineering, Technology and Science (INESC TEC), 4200-465 Porto, Portugal

**Keywords:** gait analysis, gait parameters, IMU, inertial sensors, orientation-invariant, sensor fusion

## Abstract

Inertial sensors can potentially assist clinical decision making in gait-related disorders. Methods for objective spatio-temporal gait analysis usually assume the careful alignment of the sensors on the body, so that sensor data can be evaluated using the body coordinate system. Some studies infer sensor orientation by exploring the cyclic characteristics of walking. In addition to being unrealistic to assume that the sensor can be aligned perfectly with the body, the robustness of gait analysis with respect to differences in sensor orientation has not yet been investigated—potentially hindering use in clinical settings. To address this gap in the literature, we introduce an orientation-invariant gait analysis approach and propose a method to quantitatively assess robustness to changes in sensor orientation. We validate our results in a group of young adults, using an optical motion capture system as reference. Overall, good agreement between systems is achieved considering an extensive set of gait metrics. Gait speed is evaluated with a relative error of −3.1±9.2 cm/s, but precision improves when turning strides are excluded from the analysis, resulting in a relative error of −3.4±6.9 cm/s. We demonstrate the invariance of our approach by simulating rotations of the sensor on the foot.

## 1. Introduction

Objective measurement of gait is fundamental for human motion analysis, with applications in clinical research, sports, rehabilitation, health diagnosis, and others [1]. The traditional approach for quantitative gait analysis, mostly based on the use of optical motion capture systems, was proven to be clinically relevant, however, these systems are often restricted to the laboratory setting [2]. In the clinical setting, visual observation, questionnaires or simple functional tests are commonly employed [2]. Although these evaluations require simple instruments and are easy to perform, subtle changes in spatio-temporal gait parameters (e.g., associated with geriatric syndromes like falls [3], cognitive impairment [4], or frailty [5]) can easily go undetected [2].

Inertial sensor-based gait analysis has become highly attractive in the past years, and constitutes a promising approach to assist clinical decision making for gait-related disorders in ageing [2]. Gait parameters such as gait speed, stride length, cadence, swing width, or foot clearance, can be obtained from the analysis of inertial sensor data [6,7,8,9], offering a cheaper and unrestricted alternative to gait analysis that may fit either assessment in clinical settings or continuous monitoring in daily life activities [2,10]. However, inertial sensors are susceptible to noise, and complex algorithms are needed to reliably measure gait parameters [11].

To handle errors, studies typically exploit the cyclic nature of gait and the hypothesis that the velocity of the sensor—when placed on the foot—is zero when the foot is in contact with the ground (i.e., at some point in stance) [12]. Zero velocity intervals (ZVIs) are then used to improve the methods for estimating sensor orientation [6,8,13] and reconstructing displacement [6,7,8,14,15,16]—required to evaluate spatio-temporal parameters of walking.

Even though the orientation of the sensor relative to the Earth frame can be obtained using appropriate sensor fusion methods [17,18], the orientation of the sensor relative to the movement direction or relative to the body part, is typically unknown. In some situations, for instance, when a smartphone is used to monitor users’ motion, the sensor orientation and position relative to the body may be changing over time, which requires specific methods to identify the users’ motion direction with respect to the sensor. Methods based on the ellipsoidal shape of horizontal components of acceleration [19], or based on sinusoidal approximations of the acceleration data [20] have been proposed in the literature, with direct applications on pedestrian dead reckoning. When the sensor is placed on the feet, other techniques can be used. Falbriard et al. [21] proposes an automatic calibration process, in which the Principal Component Analysis (PCA) is used to find the foot medio-lateral axis based on the angular rates acquired during gait. In [11], the foot orientation vector is estimated using a particle filter. Although [11,21] claim that the methods they propose enable the use of sensors in a robust and reproducible manner, reproducibility is not assessed by the authors.

Most studies assume that at least one sensor axis is aligned with the body—typically the medio-lateral axis when the sensor is placed on the feet. Based on this assumption, the medio-lateral angular rate and/or forward-anterior acceleration can be directly determined, and used to detect gait events [8,15]. Sagittal foot angle can also be obtained [14], from which heel and toe clearance metrics can be calculated [9,22]. The inertial coordinate system at the beginning of each gait cycle can be used as a base coordinate system (aligned with the medio-lateral and forward-anterior axis of the body), relative to which foot orientation and trajectories can be determined [15,16]. Besides assuming a known configuration, the extent to which algorithms depend on the precise alignment of the sensors has not yet been investigated.

Another approach for gait analysis relies on data-driven procedures. These methods require training in a relevant dataset, using features extracted from inertial sensor data [23] or, in case of using sufficiently deep architectures, regressing directly against raw sensor data [24]. In both cases, generalization is possible only if sufficient and representative data are given to the model, allowing it to learn from the data. Studies that employ data-driven approaches train their models without considering multiple sensor orientations [23,24], so their final models may not be robust to differences in orientation. However, assuming precise alignment of the sensors on the body seems unrealistic in practice.

In this paper, we approach the topic of orientation-invariance explicitly. We introduce an orientation-invariant gait analysis approach relying on inertial sensors placed on the shoes and validate results in a group of young adults, using an optical motion capture system as reference. We propose a method to quantitatively evaluate invariance to differences in orientation and demonstrate it by simulating rotations of the sensor on the foot. Experiments and results are presented in this work, together with a critical interpretation; findings are described considering the research problem being addressed and past work within the topic.

## 2. Materials and Methods

### 2.1. Wearable Sensors

**Figure 1:**
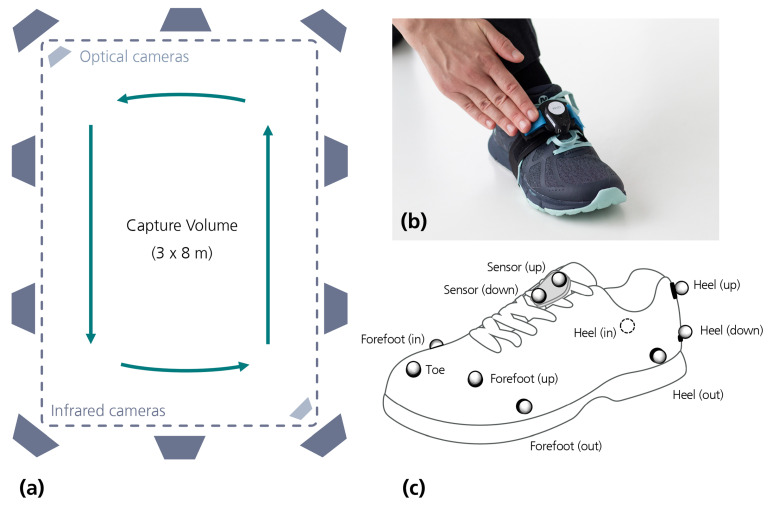
Wearable sensors and reference system: (**a**) Capture volume and camera installation. (**b**) Sensor placement. (**c**) Marker placement.

Acceleration and angular rate were measured using two inertial measurement units (IMUs). The IMUs were developed in our lab, and incorporated a 32-bit Arm Cortex M4F processor (Nordic nRF52). The device was equipped with a tri-axial gyroscope and a tri-axial accelerometer (Bosch BMI160), and communicated with a computer via Bluetooth^®^ Low Energy, enabling data collection at a sampling rate of 100 Hz. Sensors were placed on the shoes, near the foot instep, as shown in Figure 1.

### 2.2. Reference System

As a reference system, we used an optical motion capture system (Vicon, Oxford Metrics). It consisted of 10 infrared cameras (Vicon Vero v2.2), plus 2 optical cameras (Vicon Vue), installed as illustrated in Figure 1, which resulted in a capture volume of around 3 per 8 m. Optical markers were placed on participants’ shoes as illustrated in Figure 1.

The trajectories of the markers were captured at 100 Hz and post-processed using Vicon Nexus (Vicon Motion Systems Ltd., Version 2.10.1). Post-processing included automatic marker labelling, manual marker swapping correction and gap filling operations, using the methods of spline, rigid body and pattern fill available in Vicon Nexus.

After post-processing, gait parameters were automatically extracted from trajectories using a Python routine. The pipeline started with the identification of steady periods to segment signals into strides, which involved the application of thresholds to the velocity of the markers. It was followed by horizontal plane correction, where the normal (vertical) vector corresponded to the second principal axis obtained using Principal Component Analysis (PCA); PCA was applied to the position vectors–obtained from the heel (down) marker–formed between two successive steady states. Before determining gait events and calculating gait parameters, trajectories were low pass filtered using a zero-lag bidirectional first order Butterworth filter (cutoff of 20 Hz).

Initial foot contact (FC) and foot off (FO) were automatically detected, using as reference the trajectories from the markers on the heel (down) and toe. The FC instant was considered a minimum in heel vertical velocity [25], whereas the FO event was considered a maximum in vertical toe acceleration [26]. Temporal gait metrics—*stride, swing and stance duration*—were calculated as defined in [14]. *Cadence* was obtained as the inverse of stride duration, converted to the units of steps per minute.

**Figure 2:**
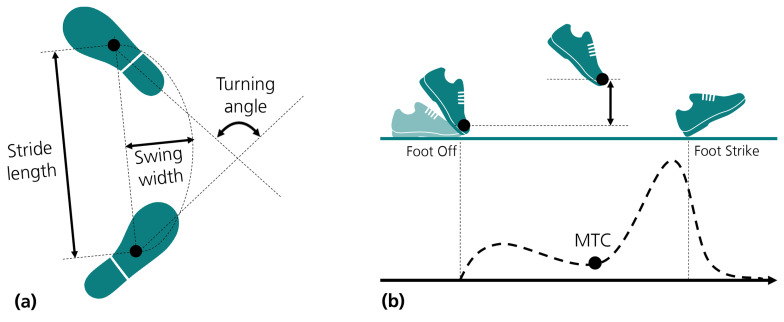
Illustration of spatial gait parameters: (**a**) Stride length, swing width and turning angle. (**b**) Minimum toe clearance (MTC).

The trajectories of the heel (down), toe and sensor centroid were used to evaluate spatial parameters, as illustrated in Figure 2: (i) *Stride length* (SL) was described as the linear distance obtained between two successive horizontal mid-stance sensor positions (as defined in [27]); (ii) *Turning angle* was defined as the angle (yaw) between two successive horizontal mid-stance foot vectors that were obtained from the positions of the heel and toe at mid-stance; (iii) *Swing width* (SW) was considered the maximum lateral excursion of the feet during swing, corresponding to the maximum-size vector perpendicular to the stride length direction; (iv) *Minimum toe clearance (MTC)* was obtained directly from toe trajectories, considered as the minimum peak vertical displacement during swing, to which the toe height at toe off was subtracted. *Gait speed* was obtained by dividing stride length by its corresponding stride duration.

All parameters were reported per stride, and calculated individually for each foot side.

### 2.3. Data Collection

A convenient sample of 26 healthy young adults (average age of 29.2±5.3 years, 13 males and 13 females) participated in data collection activities. After calibrating Vicon cameras and preparing the system for data acquisition, we placed the sensors and markers on the shoes, as shown in Figure 1. This was followed by a short static subject calibration trial. Afterwards, we asked the participant to do three consecutive laps, during which Vicon and IMU data were collected simultaneously. In each lap, participants were requested to walk straight along the length of the capture volume (i.e., along the 8 m) and use the width of the capture volume to turn (as illustrated in Figure 1). Participants repeated the walking trials in both directions—clockwise and counterclockwise—walking at comfortable, slower and faster speeds (self-selected), which resulted in 6 acquisitions per participant. All conditions were then repeated once. The study received approval by the Ethical Committee of the University of Porto (81/CEUP/2019) and all participants provided written informed consent.

### 2.4. IMU Data Processing

The following sections describe the methods for IMU data processing, inspired by typical gait analysis routines—including the stages of zero velocity detection, orientation estimation, double integration, events detection, and gait parameters estimation. The processing pipeline included evaluation of different approaches for orientation estimation and double integration. Every stage was formulated using an orientation-invariant approach.

#### 2.4.1. Zero Velocity Detection

Zero velocity intervals (ZVIs) were detected using the angular rate energy detector [28]. According to [28], angular rate provides rich information concerning the detection of ZVIs. Compared to other methods (e.g., the acceleration magnitude detector), the angular rate energy detector achieved the highest performance [28].

To calculate the energy of the angular rate magnitude, we used a sliding window with 0.15 s. The size of the window was experimentally set to ensure an appropriate energy result, i.e., not too smoothed, nor too noisy. The window size of 0.15 s resulted in a good compromise that could adapt to all walking speeds considered. To determine ZVIs, a threshold was applied to the energy of the angular rate magnitude. The threshold was calculated with basis on the average of the energy, to ensure that differences in walking speed (reflected as different signal amplitudes) would be considered. The threshold was experimentally set at 1/8 of the average, to ensure that all strides in all velocities would be captured by the method.

After roughly detecting ZVIs, we proceeded with a dynamic adjustment of the intervals. For each ZVI, we did a threshold refinement search: starting with a very low threshold, we have progressively increased it (in steps of 1/20 of the ZVI energy range), until a minimum interval size was obtained. The minimum interval size was set at 0.1 s, unless an interval lower than 0.1 s was registered in the trial. Using this process, we ensured a confident detection of all strides and, additionally, that all ZVIs were refined to potentially include only zero velocity instants. The remaining intervals, i.e., the moving intervals, were used to evaluate movement in the subsequent processing stages.

#### 2.4.2. Orientation Estimation

Orientation, or attitude, of the sensor relative to the global frame of reference was expressed using quaternions. The global frame of reference was defined by two perpendicular horizontal axes (x and y)—arbitrarily set—and a vertical axis, z, pointing to the sky. To obtain quaternions, we tested three methods: gyroscope integration and two complementary filters (CFs)—Madgwick and Euston.

##### Gyroscope Integration

The gyroscope integration method takes advantage of the knowledge of the moving and not-moving intervals. When the sensor is not moving (i.e., during a ZVI)—when all measured accelerations are due to the Earth’s gravity acceleration—the accelerometer signal is used to estimate sensor inclination. The vector $g_{s}=\left[{\bar{a}}_{x},{\bar{a}}_{y},{\bar{a}}_{x}\right]$, defined with the average acceleration values measured while the sensor is not moving, corresponds to the z-axis in the global frame of reference, i.e., the gravity axis. The horizontal axis—y-axis, perpendicular to gravity—is then defined arbitrarily. The initial quaternion is obtained from gravity and the horizontal vector, using the tri-axial attitude determination (TRIAD) algorithm [29].

The initial quaternion is updated each time the foot is in contact with the ground. During the intervals when the foot is moving, the quaternion q(t) is updated resorting to the integration of angular rate ω(t) measured by the gyroscope, as described in [8,13]. This is performed as defined by Equations (1) and (2):

(1) $$\dot{q}\left(t\right)=\frac{1}{2}q\left(t-\Delta t\right)⊗p\left(\omega \left(t\right)\right)$$

(2) $$q\left(t\right)=\frac{q\left(t-\Delta t\right)+\dot{q}\left(t\right)\Delta t}{||q\left(t-\Delta t\right)+\dot{q}\left(t\right)\Delta t\left|\right|}$$

where $\dot{q}\left(t\right)$ is the quaternion derivative and Δt is the sampling interval. The function p(.) denotes the quaternion representation of a vector and the ⊗ operator represents the quaternion product.

##### Madgwick CF

In Madgwick [30], the quaternion derivative $\dot{q}\left(t\right)$ used in Equation (2) is replaced by a corrected estimate $\dot{\hat{q}}\left(t\right)$ that incorporates orientation information provided by the accelerometer. The method is based on the calculation of the gradient descent, as shown in Equation (3).

(3) $$\dot{\hat{q}}\left(t\right)=\dot{q}\left(t\right)-\beta \frac{\Delta \varepsilon (t)}{∥\Delta \varepsilon (t)∥}\;\;\mathrm{with}\;\Delta \varepsilon \left(t\right)=J{\left(\varepsilon (t)\right)}^{T}\varepsilon \left(t\right)$$

where *J* denotes the Jacobian, β is the filter gain and ε(t) denotes the error term, obtained by subtracting the accelerometer measurement in the sensor frame $a_{s}\left(t\right)$ to the theoretical gravity vector in sensor coordinates $g_{s}\left(t\right)$ (obtained by transforming gravity in global coordinates to sensor coordinates). β can be defined as $\beta =\sqrt{3/4}\;{\bar{\omega }}_{max}\pi /180$, where ${\bar{\omega }}_{max}$, expressed in degrees, represents the maximum gyroscope measurement error (mean zero gyroscope measurement error).

The Madgwick filter is applied at all instants of the signal (moving and not moving intervals) considering as basis the initial quaternion determined using TRIAD, as described previously.

##### Euston CF

The explicit complementary filter, also known as Euston filter, was implemented as described in [13,18]. In Euston, instead of replacing the value of the quaternion, the measured angular rate ω(t) is replaced by a corrected angular rate signal, resulting in the following filter dynamics:

(4) $$\dot{\hat{q}}\left(t\right)=\frac{1}{2}\hat{q}\left(t-\Delta t\right)⊗p\left(\omega (t)+\delta \left(t\right)\right)$$

in which the error term δ is obtained following Equations (5) and (6), where the term e(t) describes the angular mismatch between theoretical ($g_{s}\left(t\right)$) and measured ($a_{s}\left(t\right)$) direction of gravity [18].

(5) $$e\left(t\right)=\frac{g_{s}\left(t\right)}{∥g_{s}\left(t\right)∥}\times \frac{a_{s}\left(t\right)}{∥a_{s}\left(t\right)∥}$$

(6) $$\delta \left(t\right)=k_{P}e\left(t\right)+k_{I}\int e\left(t\right)dt$$

The Euston filter has two adjustable parameters, the proportional gain $k_{P}$—to separate low- and high-frequency estimates of orientation—and the integrator gain $k_{I}$—to compensate for gyroscope bias [13,18]. Similarly to the Madgwick filter, we apply Euston to all instants of the signal, considering as basis the initial quaternion determined using TRIAD [29].

#### 2.4.3. Double Integration

After obtaining orientation quaternions, q(t), we calculate linear acceleration in global coordinates, hereinafter represented as a(t) for simplicity. To that purpose, we first estimate the value of the gravity vector in global coordinates, $g_{w}=\left[0,0,{\bar{a}}_{zv}\right]$, where ${\bar{a}}_{zv}$ is the average acceleration magnitude measured during ZVIs. Linear acceleration is then obtained as shown in Equation (7).

(7) $$a\left(t\right)=q\left(t\right)⊗p\left(a_{s}\left(t\right)\right)⊗q^{-1}\left(t\right)-g_{w}$$

where $a_{s}\left(t\right)$ represents raw acceleration, as measured by the sensor.

To obtain displacements, we integrate linear acceleration two times. On the first integration, an estimate of velocity, $\hat{v}\left(t\right)$, is obtained. Integrals are computed using the Trapezoidal Rule, as shown in Equation (8).

(8) $$\hat{v}\left(t\right)\approx \sum_{i}\frac{a_{i}+a_{i-1}}{2}\Delta t$$

To bound the errors, two different methods—linear dedrifting and direct and reverse integration—are employed, as we explain next. After obtaining trajectories, a novel approach to correct the final vertical position (assuming walking on a flat surface) is tested. The method rotates the trajectories so that the final height of each stride is zero. To that purpose, a rotation quaternion is calculated, using as basis the angle with the horizontal plane at the end of the stride and the rotation vector calculated as the cross product between the vertical axis and the stride displacement vector. The rotation quaternion is used to rotate trajectories within each moving interval.

##### Linear Dedrifting

Double integration is performed between ZVIs, on a stride-by-stride basis. To fulfil the zero-velocity assumption—on which moving intervals are bounded by zero velocity instants—a linear drift function ($d_{v}\left(t\right)$) is estimated and subtracted from the estimated velocity, as described in [14] and shown in Equation (9).

(9) $$v\left(t\right)=\hat{v}\left(t\right)-d_{v}\left(t\right)$$

Trajectory s(t) is obtained by integrating again velocity v(t).

##### Direct and Reverse Integration

The direct and reverse integration method fuses the regular integral with a time-reversed integral so that the boundary conditions, in this case, the zero-velocity conditions, are satisfied in the initial and final values of the integral [15,16]. The result of direct ($v_{\to }\left(t\right)$) and reverse ($v_{\leftarrow }\left(t\right)$) integration is combined using a sigmoid weighting function (w(t)), as shown in Equation (10).

(10) $$v\left(t\right)=\left(1-w\left(t\right)\right)v_{\to }\left(t\right)+w\left(t\right)v_{\leftarrow }\left(t\right)$$

The sigmoid w(t), specified in Equation (11), is shaped using the steepness parameter, η, and the inflection point, $t_{i}$, defined between the temporal bounds $t_{n}$ and $t_{n+1}$ of each moving interval. To define $t_{i}$, a proportion $\alpha_{i}$, between 0 and 1, of the moving interval is used.

(11) $$w\left(t\right)=\frac{h\left(t\right)-h\left(t_{n}\right)}{h\left(t_{n+1}\right)-h\left(t_{n}\right)}\;\;\mathrm{with}\;h\left(t\right)={\left(1+\exp\left(-\frac{t-t_{i}}{\eta }\right)\right)}^{-1}$$

Position is estimated by integrating velocity.

#### 2.4.4. Events Detection

To detect gait events avoiding the need of determining angular rate or acceleration in body coordinates (where the alignment of the sensor on the body would need to be known [8,15,16]), we used acceleration magnitude and the vertical component of acceleration (in global coordinates).

**Figure 3:**
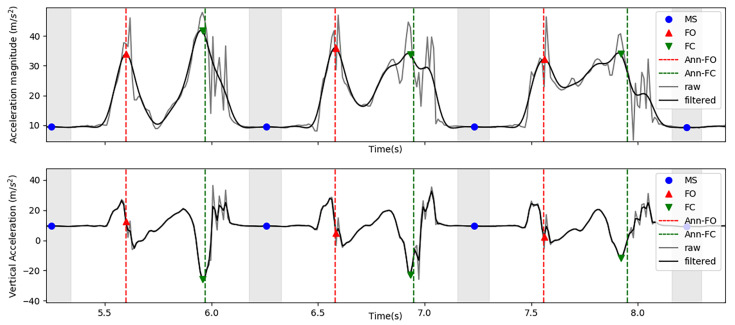
Events detection from low pass filtered acceleration magnitude and low pass filtered vertical acceleration (*FO*—Foot off; *FC*—Initial foot contact; *MS*—Mid-stance; *Ann-FO*—Annotated FO; *Ann-FC*—Annotated FC).

We observed that FO events can generally be found in an acceleration magnitude perturbation before swing. To approximate this instant, we filtered acceleration magnitude using a zero-lag bidirectional 2nd order Butterworth low-pass filter with a cutoff of 7 Hz. The cutoff frequency was experimentally chosen to ensure that (i) the perturbations were smoothed resulting in a single peak value, and (ii) the resultant peak approximates the acceleration magnitude perturbation where annotated FO events are observed. The FO event was considered the first maximum peak above the average of the moving interval appearing between two ZVIs (i.e., the first value above the average surrounded by two values with lower magnitude), as illustrated in Figure 3.

FCs are detected between FOs and the beginnings of the next ZVIs. FCs were considered the absolute minimum of vertical acceleration measured between these two instants (Figure 3). Before detecting FC events, vertical acceleration was low-pass filtered using a zero-lag bidirectional 1st order Butterworth filter with a cutoff of 30 Hz. The cutoff frequency was experimentally chosen to ensure the attenuation of high frequency noise that could hinder the detection of FC events.

#### 2.4.5. Gait Parameters Estimation

After calculating orientation, position and determining FO and FC events, temporal and spatial parameters were estimated for each gait cycle *n*. Temporal parameters—stride, swing and stance duration—were determined as defined in [14]. Cadence was obtained as the inverse of stride duration, expressed in steps per minute. Spatial parameters (illustrated in Figure 2) were calculated using information of moving intervals, defined by the temporal bounds of $t_{n}$ and $t_{n+1}$. To estimate SL and SW, we used trajectories on the horizontal plane, $s_{xy}$, as determined by Equations (12) and (13), where ${\vec{s}}_{n}\left(t\right)$ represents a displacement vector relative to the final stride position at $t_{n+1}$, obtained as $s_{xy}\left(t_{n+1}\right)-s_{xy}\left(t\right)$). In Equation (13), the symbol *∠* denotes the angle between two vectors.

(12) $${SL}_{n}=∥{\vec{s}}_{n}\left(t_{n}\right)∥$$

(13) $${SW}_{n}=\max_{t\in \{FO_{n}:FC_{n+1}\}}∥{\vec{s}}_{n}\left(t\right)∥\sin(∠\left({\vec{s}}_{n}\left(t_{n}\right),\;{\vec{s}}_{n}\left(t\right)\right)$$

Gait speed was obtained by dividing SL by its corresponding stride duration.

**Figure 4:**
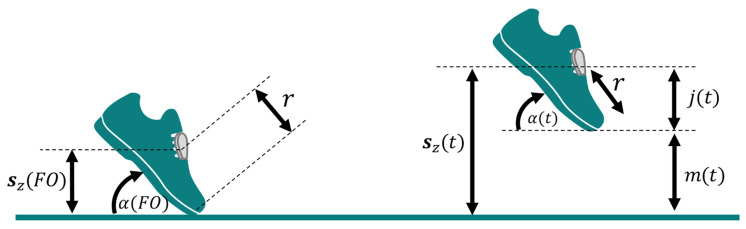
Variables involved in the calculation of toe trajectory.

To calculate MTC, we used a method inspired on the work by Kanzler, C. [9]. To estimate toe trajectory, we have first estimated the distance between the sensor and the toe, *r*, using as a basis the angle produced by the foot at FO, $\alpha (FO_{n})$, in each gait cycle *n*, as illustrated in Figure 4.

To obtain the angle α(t), we did a series of vector transformations. First, we have converted the vertical vector [0,0,1] to sensor coordinates, using the quaternion at the beginning of the moving interval, i.e., at the foot flat at $t_{n}$. Then, we transformed the resultant vector back to global coordinates using the quaternion at FO. This vector, ${\vec{v}}_{w}$, was used to estimate the medio-lateral vector, ${\vec{l}}_{w}$, using the cross product between ${\vec{v}}_{w}$ and [0,0,1]. The forward vector, ${\vec{f}}_{w}$, was calculated using the cross product between [0,0,1] and ${\vec{l}}_{w}$, which was then converted back to sensor coordinates using the quaternion at $t_{n}$. This vector, ${\vec{f}}_{s}$, parallel to the ground at foot flat and pointing forward towards the toes, was used to estimate the angle α(t), as depicted in Equation (14).

(14) $$\alpha \left(t\right)=∠\left(\left[0,0,1\right],\vec{v}\right)-\alpha \left(t_{n}\right)\;\;\mathrm{with}\;\vec{v}=q\left(t\right)⊗p\left({\vec{f}}_{s}\right)⊗q{\left(t\right)}^{-1}\;\mathrm{and}\;t\in \left[t_{n}:t_{n+1}\right]$$

The distance between the sensor and the toe (*r*) was obtained by the average of the values determined in each stride *n*, as shown in Equation (15).

(15) $$r=\frac{1}{N}\sum_{n=1}^{N}\frac{s_{z}\left(FO_{n}\right)}{sin(\alpha \left(FO_{n}\right))}$$

where *N* is the total number of strides and $s_{z}\left(t\right)$ represents the z component of the trajectory of the sensor (i.e., its vertical displacement). MTC was considered the minimum peak vertical toe displacement measured during the swing phase of walking. This vertical displacement, m(t), was estimated as shown in Equation (16), where r×sin(α(t)) represents the vertical distance between the sensor and the toe (shown as j(t) in Figure 4).

(16) $$MTC_{n}=\min_{t\in \{FO_{n}:FC_{n+1}\}}m\left(t\right)\;\;\mathrm{with}\;m\left(t\right)=\left\{\begin{array}{cc} s_{z}\left(t\right)-r\times sin\left(\alpha \left(t\right)\right) & \mathrm{if}\;\alpha (t)>0\\ s_{z}\left(t\right)+r\times sin\left(\alpha \left(t\right)\right) & \mathrm{otherwise} \end{array}\right.$$

To calculate turning angles, we converted an arbitrary horizontal vector (e.g., the vector [0,1,0]) to sensor coordinates, using as basis the quaternions estimated at $t_{n}$ and at $t_{n+1}$. The resultant vectors represented the orientation of the sensor in the horizontal plane, so that the angle between these two vectors corresponded to the turning angle.

### 2.5. Experiments

One hundred and sixty of the collected samples were post-processed using Vicon Nexus, and used as reference for IMU-based gait analysis evaluation. To avoid overfitting, we split data into development and validation sets. Approximately 30% of the samples, from uniquely randomly selected users, were included in the development set, and used for algorithm design, debug and optimization. The remaining 70% were used for validation and orientation-invariance proof. The resulting dataset split is shown in Table 1.

To compare gait parameters extracted from the IMU with those extracted from Vicon, systems were first synchronized. To this purpose, we used the cross-correlation between acceleration magnitude—obtained from the IMU—and the centroid of the sensor markers—obtained after deriving the centroid trajectory two times; the maximum cross-correlation was used to compensate for the time-shift between data sources, as in [6].

A tolerance of 0.1 s was employed to classify FC events (and its corresponding stride gait parameters) as true positive cases. Only the strides whose FC time was consistent with those obtained with Vicon were considered for comparison. Reference strides without any corresponding IMU-derived candidate were classified as not detected.

#### 2.5.1. Parameter Tuning and Algorithm Selection

The development set was used to tune parameters, and select the most reliable methods for orientation estimation and double integration. As shown in Section 2.4.2, the possible orientation estimation methods were the Madgwick CF, the Euston CF and the gyroscope integration, which could be used in combination with two possible double integration methods: the direct and reverse integration, and the linear dedrifting—both presented in Section 2.4.3. Additionally, the horizontal correction method presented in Section 2.4.3 could or not be employed. Each method—with the exception of gyroscope integration and linear dedrifting—included a set of parameters (also detailed in Section 2.4.2 and Section 2.4.3) that needed to be optimized in view to improve the performance of the gait analysis method. Using a grid-search approach, all possible combination of methods (i.e., orientation estimation and double integration methods) and a set of candidate parameters could be tested, where the number of resultant combinations depended on the number for parameters tested. For this reason, parameter tuning was first performed using a coarse grid of parameters—i.e., a small amount of candidate parameters covering a wider range of values. The most promising combinations of parameters and methods were used to define a finer grid that considered candidate parameters defined in the neighbourhood of the best parameter configurations. Parameters tested within coarse (resulting in 140 combinations) and fine grid-search (resulting in 105 combinations) are shown in Table 2.

To select the best combination of parameters and methods, we calculated the root mean square error (RMSE) between IMU-derived and reference gait parameters. The RMSE of each parameter was normalized by the average of the reference, and then averaged to obtain a single score per configuration. The minimum normalized RMSE defined the most appropriate set of parameters and methods.

#### 2.5.2. Instrument Comparison and Validation

We compared gait parameters extracted from the IMU with those extracted from Vicon using the validation set. For each cycle, we estimated the difference between IMU-derived and reference gait parameters. Accuracy (mean of relative and absolute error) and precision (standard deviation of relative and absolute error) were reported for each parameter. Agreement between the two instruments was assessed using 95% limits of agreement, as introduced by Bland Altman [31]. Data were assessed for normal distribution using Shapiro–Wilk tests, to decide for the use of parametric or non-parametric tests. Correlation between instruments was calculated using the correlation coefficients of Pearson ($r_{p}$)—in case of normal distribution—or Spearman ($r_{s}$)—when data could not be assumed to be normally distributed. We have also reported RMSE and equivalence tests using an equivalence zone of ±5% of the average of the metric. Equivalence tests were based on Paired *T*-test (*T*)—for parametric—or Wilcoxon signed-rank test (*W*)—for non-parametric.

To validate results in a scenario where only straight walking is considered for gait assessment (as required to assess several gait disorders [32]), we repeated validation tests without including turns. For this purpose, a turning stride was considered a stride where the turning angle (as measured by the reference system) was above 20 degrees (as in [7]).

A significance level (*p*-value) of 5% was used to evaluate results.

#### 2.5.3. Orientation Invariance

To test for orientation invariance, we simulated multiple rotations of the IMU on the shoes. For that purpose, we sampled uniform random rotations (quaternions), as suggested by Shoemake, K. [33], and used those quaternions to synthetically rotate raw inertial sensor data. To evaluate the performance of the system when IMUs were placed at random rotations, we compare gait parameters extracted from the original sensor orientation with those extracted from a rotated version of the sensor. To quantify differences, we calculated the Root Mean Square Deviation (RMSD), correlation (using Pearson-parametric-or Spearman-non-parametric) and equivalence tests using a stricter equivalence zone of ±1% of the average of the metric. Equivalence tests were based on Paired *T*-test (*T*)—for parametric—or Wilcoxon signed-rank test (*W*)—for non-parametric. To choose an appropriate test, samples were first tested for normal distribution using Shapiro–Wilk; non-parametric tests were chosen in case of non-normal distribution. A significance level (*p*-value) of 5% was used to evaluate results.

## 3. Results

### 3.1. Algorithm Selection and Parameter Tuning

The most promising combinations of parameters and methods were defined by the results of the coarse grid-search. We selected the best performing combinations considering the minimum normalized RMSE achieved by each candidate method. The best combinations in coarse grid-search were: (i) the Madgwick CF (${\bar{\omega }}_{max}=2.0$) combined with direct and reverse integration (η=0.2, $\alpha_{i}=0.6$) or with linear dedrifting methods; (ii) the Euston CF ($k_{P}=0.2$, $k_{I}=0$) combined with direct and reverse integration (η=0.2, $\alpha_{i}=0.6$); and (iii) the gyroscope integration combined with linear dedrifting, all with active horizontal correction. The second optimization was performed using a finer grid of parameters (shown in Table 2), defined on the vicinity of the best performing combinations. The resulting best combination of methods and parameters was the Euston CF ($k_{P}^{opt}=0.15$, $k_{I}^{opt}=0$) with direct and reverse integration ($\eta^{opt}=0.25$, $\alpha_{i}^{opt}=0.55$) and active horizontal correction. The RMSE values obtained in the development set using this parameter configuration are shown in Table 3.

### 3.2. Validation

The best combination of methods and parameters was used to generate results for validation. In the analysis, 7015 strides out of 7142 (i.e., approximately 98.2%) were included, meaning that only 127 strides (i.e., 1.8%) were classified as not detected. FC events were detected with an average relative error of −0.01±0.02 s and limits of agreement of −0.06 and 0.04 s. FO events were detected with an average relative error of −0.01±0.05 s and limits of agreement of −0.11 and 0.08 s. FC and FO events were detected with the same average relative error, but the dispersion of the errors in FO detection was higher, as evidenced by the standard deviation and limits of agreement.

The comparison of IMU-derived and reference gait parameters, obtained in the validation set, is shown in Table 4.

According to the classification proposed in [34], high (i.e., between 70 and 90) to very high (i.e., above 90) correlation was obtained in all variables, except MTC that had a moderate correlation ($r_{s}=0.55$). Equivalence tests revealed all metrics to be practically equivalent (with p<0.01), except MTC, considering equivalence intervals corresponding to 5% of the average of the metric. Average absolute errors in stride duration of 0.02±0.05 s were obtained, which represents an average error of two samples considering the sampling rate of 100 Hz. SL had an average relative error of −3.5±9.7 cm (Table 4).

Results excluding turns are shown in Table 5, where about 2% of the reference strides (i.e., 78 strides) are considered as not detected, resulting in the analysis of 3785 strides. Correlations between variables are high (i.e., between 70 and 90) or very high (i.e., above 90), except for MTC and turning angles where Spearman correlations are of 0.55 and 0.68, respectively, and classified as moderate. Accordingly, equivalence tests reveal all metrics to be practically equivalent (with p<0.01), except for MTC and turning angles. Average relative errors of 0.00±0.04 s and −3.9±6.2 cm were obtained for stride duration and SL, respectively (Table 5).

### 3.3. Orientation Invariance

The comparison of gait parameters extracted using the original sensor orientation and using simulated rotations is shown in Table 6. As can be observed, all parameters, except turning angle, present the same values when the sensor is rotated, i.e., all parameters have a RMSD of 0.0, except turning angle that has a RMSD of 1.5${}^{\circ }$. The correlation between turning angles extracted using the original orientation and random rotations of the sensor is very high ($r_{s}=0.99$). The performance of the system when data are synthetically rotated remains practically equivalent (equivalence tests have p<0.01 for all gait metrics, considering an equivalence interval of 1% of the average of the metric).

**Figure 5:**
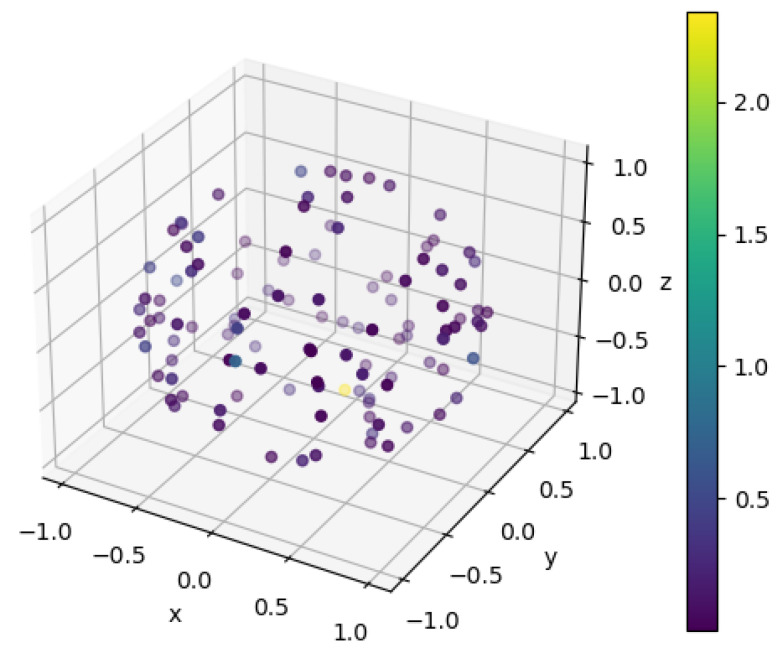
Synthetically generated rotations and their relationship with differences in measured turning angles. Colours represent the absolute difference in turning angles, presented in degrees.

Figure 5 allows further (visual) inspection of the generated rotations and their relationship with the differences in measured turning angles (the only metric that presented some differences when the sensor was synthetically rotated). As can be observed, rotations are uniformly distributed across all 3D space. The differences in turning angles are randomly distributed through the space, showing no particular tendency towards a specific region.

## 4. Discussion

In this work, we proposed an orientation-invariant gait analysis approach: sensor alignment on the foot is unknown and can be anything. We based our approach in previously studied methods, where some adaptations were introduced in view to maintaining all methods independent to differences in sensor orientation.

To this purpose, orientation-invariant signals were used. To detect ZVIs, we relied on the energy of angular rate, as in [28]. To detect gait events (FC and FO), instead of using medio-lateral angular rate and/or forward acceleration (as proposed by [8,15] or [16]), we used features from acceleration magnitude and from the vertical component of acceleration, obtained in global coordinates.

Instead of representing trajectories using the body coordinate system (as proposed by [15,16]), we evaluated trajectories in global coordinates, from which SL and SW could be estimated. However, to calculate MTC, we estimated a medio-lateral and a forward axis, that were defined with basis on the medio-lateral rotation produced by the foot when moving from foot-flat to FO. These axes were required to estimate the angle α(t)—as shown in Equation (14)—and could be used to obtain sensor data in body coordinates—similar to the use of PCA, as proposed by Falbriard et al. [21].

The proposed approach was tested in a group of young adults, without any visible gait disorder, using an optical motion capture system as reference. We tested multiple orientation estimations and double integration approaches. Based on the results achieved on the development set, we selected the Euston CF ($k_{P}^{opt}=0.15$, $k_{I}^{opt}=0$), combined with direct and reverse integration ($\eta^{opt}=0.25$, $\alpha_{i}^{opt}=0.55$) with active horizontal correction. With an $\eta^{opt}$ of 0.25, the sigmoid used in direct and reverse integration (Equation (11)) approximates the linear shape. Yet, the results achieved with the linear and reverse integration were better than the results achieved with the linear dedrifting method. According to [13,18], the $k_{I}^{opt}=0$ used in Euston CF is an appropriate choice due to the short integration times (below 5–10 min) and the slow dynamics of the movements involved. The horizontal correction mechanism allowed for a compensation of the final vertical position achieved in each stride, assuming walking on a flat surface. Although it improved the results in our study, the method can only be applied when walking on a flat surface, and cannot be generalized to walking in inclined surfaces or stairs.

In [13], the same orientation estimation and double integration methods were benchmarked, using a dataset comprising 20 healthy subjects (between 16 and 80 years old), all using the same shoe model and walking straight at multiple speeds in a path with 10 m. According to the authors, the best performing orientation estimation method (evaluated by the angles obtained in each axis) was Madgwick CF (${\bar{\omega }}_{max}=3.04$), followed by gyroscope integration and Euston CF ($k_{P}=0.0046$, $k_{I}=0$); but the performance of the three methods did not differ much. For double integration, the direct and reverse integration (η=0.08, $\alpha_{i}=0.6$) was the best performing method, which is also according to our results. The optimal set of parameters differed from ours, which may be explained by differences in datasets, namely due to different sensor characteristics, subjects, and protocol. While we apply orientation estimation methods at all instants of the signal, in [13] acceleration data are only used when the magnitude of the acceleration approximates the value of the gravity (i.e., the sensor is roughly static). Moreover, the criteria for evaluating methods performance differed from ours. In [13], orientation estimation methods were optimized first based on the measured angles; double integration methods were optimized after selecting the best orientation estimation method, using as criteria the error distribution of estimated velocities and clearance parameters [13]. In our study, we considered that orientation estimation methods have an impact on the performance of double integration methods, which, altogether, have an impact on all gait metrics considered. Therefore, optimizing for a single metric could penalize the performance of the other metrics. For this reason, we opted for the jointly selection of methods and the tuning of parameters based on the overall results achieved in all metrics considered.

The results achieved on the development set with the optimized set of methods and parameters is shown in Table 3. Even though optimization was performed in this set, some of the metrics differ by some units from the reference system. Table 4 shows the results on the validation set. As expected, the central tendency and the dispersion of the errors are lower on the development set where algorithm selection and parameter tuning occurred. The generalization of the method can only be effectively evaluated using the validation set, which included data from unique users never seen during the optimization process.

Using the sensor on foot instep, Mariani et al. [35] tested gait event detection using multiple candidate features (i.e., minimum, maximum or zero-crossing) and multiple signals—including signals that are independent of IMU orientation, namely acceleration magnitude and the derivative of angular rate magnitude. According to their results, the best candidate features are observed in the acceleration magnitude signal [35], close to the instants we propose to detect FC and FO in this work. On average, FO and FC events were detected 0.01 s (i.e., one sample) before the annotations, but the dispersion of the errors was higher for FO detection. While FCs can generally be well perceived on acceleration signals (they represent an impact of the foot on the ground), TO events may be less evident, which may justify the results. Nevertheless, event detection errors are, in general, low, causing a small impact on estimated temporal parameters.

Our event detection method resulted in absolute errors between 0.02 s and 0.03 s in temporal parameters, with precision between 0.03 s and 0.05 s (as shown in Table 4 and Table 5). These errors represent average differences of just two or three samples at 100 Hz, not differing much whether we include turns or not, which demonstrates the robustness of the method. In [8], relative errors of 0.00±0.07 s, −0.01±0.04 s, and 0.01±0.07 s are obtained for stride, swing and stance duration in a group of geriatric inpatients, where events are determined based on the analysis of medio-lateral angular rate and forward acceleration, assuming a fixed alignment of the sensor on the shoes. In [16], sensors are placed on the shanks, obtaining relative errors of 0.00±0.02 s in stride duration in a group of young adults. Although the performance on temporal parameters is more or less consistent between studies, it is worth mentioning that while studies differ in subjects and protocols applied, the procedures to estimate reference gait events are also different, which hinders comparison of results. Moreover, these studies are conducted in a laboratory setting, ensuring the precise alignment of the sensors on the body. For this reason, they may not represent the results that would be achieved in a more realistic clinical setting.

In our study, relative errors of −3.5±9.7 cm and −3.1±9.2 cm/s were obtained for SL and gait speed, respectively. When we exclude turns from the analysis, the precision is improved, resulting in relative errors of −3.9±6.2 cm for SL and of −3.4±6.9 cm/s for gait speed. Additionally, RMSE values are lowered and correlations increase (see Table 4 and Table 5). These results show that increased errors in SL (reflected also as increased errors in gait speed) may be related with differences imposed by gait patterns while turning, so that evaluation of these metrics in straight walking seems more reliable. Results would possibly improve if parameter optimization was performed using straight walking exclusively, however, at the cost of making the method less robust.

In [7], relative errors of 1.3±3.0 cm and of 2.8±2.4 cm/s were obtained for SL and speed, respectively, in a group of patients with Parkinson’s Disease and age-matched elderly subjects, after discarding turning, initiation and termination cycles. In [8], relative errors of −0.3±8.4 cm were obtained for SL in a group of geriatric inpatients walking straight. Using the same dataset, Hannink et al. [24] achieved relative errors of 0.0±5.4 cm, using a deep convolutional neural network. In [16], SL and gait speed had a performance of 5.4±3.1 cm and 3.4±3.9 cm/s, considering only data from young adults obtained while walking straight. In a group of young and elderly volunteers, Mariani et al. [6] achieved performance of 1.5±6.8 cm for SL and 1.4±5.6 cm/s for speed, using a protocol that included assessment with U-turn and 8-turn. As we can see from the literature, results are highly heterogeneous and comparison of performance in a fair and robust way is not possible due to the different protocols, subjects, and reference systems employed. Yet, considering that these metrics had very high correlations with the reference system in our study, we can consider our approach appropriate to assess movement performance. This is especially true when we consider straight walking tests, where precision is improved.

Although the precision of SW is improved when turns are excluded (relative error of −0.4±4.4 cm versus 0.1±2.8 cm when turns are excluded) and RMSE is lowered (4.4 cm versus 2.8 cm), the correlation between IMU-based and reference-based SW is worse when turns are excluded. We also observe that, when we exclude turns, the average and standard deviation of the metric decreases, meaning that curves may be associated with increased SW necessary to describe the trajectory of the turn. When we exclude turns, the values of SW are more consistent (closer to the mean) and, as such, harder to correlate, which may justify the results. The same observation is also valid for turning angles where we can see that even though precision is improved (relative error of $0.9\pm 8.6^{\circ }$ versus $1.2\pm 5.1^{\circ }$), the correlation decreases when we exclude turns (see Table 4 and Table 5). In [7], relative errors of 0.15±2.13 cm and $0.12\pm 3.59^{\circ }$ are obtained for SW and turning angle, considering only straight and steady walking.

MTC is one of the metrics with highest errors, considering their relation to the average of the metric. Relative errors of −0.2±0.8 cm and −0.4±0.7 cm are obtained for MTC when turns are included and excluded, respectively; we observe a small improvement in precision and RMSE when turns are excluded, which again, highlights the challenging conditions possibly imposed by the turns.

In [22], a method is proposed to determine toe trajectories that requires the size of the shoe as an input; using this method, authors achieved relative errors of 1.3±0.9 cm in MTC, in a group of healthy adults walking straight, where only steady walking was included in the analysis. In [9], prior information about shoe dimensions was not required, and relative errors of 1.7±0.7 cm were obtained in a group of young, mid-age and old subjects, where conditions for straight or turning walking are not specified. Although the reported relative errors are similar to our results, higher correlations (0.91) are documented by the authors [9]. However, to achieve these results, Kanzler et al. [9] employ a correction that adjusts the amplitude of toe clearance trajectories—possibly penalizing generalization of the method. Moreover, authors discuss that the assumption of the rigid shoe model may not be realistic due to the bending of the shoe at FO, which may also constitute a source of errors in our approach. These errors, combined with a poor estimation of sensor-to-toe distance and errors imposed by the reference—due to a toe marker that is not placed precisely at the tiptoe—can possibly justify the results.

All gait metrics extracted using the IMU present moderate, high or very high correlation with the reference system. Metrics are also practically equivalent, except for MTC and for turning angle when turns are excluded (Table 4 and Table 5). Based on these results, we can state that, overall, there is a good agreement between both systems, which denotes the potential of the solution. Sensor wireless capabilities, combined with a flexible alignment on the foot—fostered by the orientation-invariant approach—makes the proposed solution promising for use at clinics and ambulatory settings.

Although the reference system used in this study is currently considered the gold standard for gait analysis [6,7,9,15,16], possible errors can be introduced by the reference. For instance, due to errors in markers labelling or gap filling operations, trajectories of the markers may not always reliably replicate the actual trajectories performed. Additionally, detection of events from trajectories may also display errors that can impact temporal parameters used as reference [26].

Despite our efforts to make the process completely invariant to differences in sensor orientation, we can observe some differences in turning angles when we synthetically rotate sensor data (see Table 6). These differences have no apparent correlation with the generated rotations, as can be visually confirmed in Figure 5. Moreover, rotations were uniformly sampled to ensure a uniform distribution along all possible 3D transformations (Figure 5), which avoided bias on observations—i.e., ensuring that the method is truly invariant to changes in sensor orientation, no matter how it is oriented relative to the feet. The differences in turning angle, not observed in any other metric extracted, may be explained by the sensor fusion process employed to estimate sensor orientation relative to the global frame. While the estimation of the vertical axis employs corrections based on measured acceleration values, in the horizontal plane no correction mechanisms can be employed, and only integration of angular rates are used to estimate changes in orientation (or heading) over time. This may lead to cumulative error propagation due to the integration process, which may differ depending on sensor orientation: different sensor orientations may lead to distinct error propagation profiles. Given the random nature of the noise properties of gyroscope measurements [36], no particular tendency for an increased error towards a specific rotation can be observed. All gait parameters extracted using synthetically rotated data remained practically equivalent (see Table 6), which demonstrates the invariance of the method to differences in orientation. Based on these results, we can state that our method does not require careful alignment of the sensor on the foot, which may increase trust and potentially simplify the data acquisition process in the context of clinics.

Future work should address spatio-temporal gait analysis in specific groups (e.g., older adults or groups with a specific pathology), so that validity and robustness of the method to different walking pattern characteristics may be assessed, demonstrating its possible application in real scenarios.

## 5. Conclusions

Foot-worn inertial sensors were used to evaluate a comprehensive set of spatio-temporal gait metrics in a group of young adults. To avoid restrictions on sensor alignment, we proposed an orientation-invariant gait analysis approach, and assessed its performance using an optical motion capture system as reference. Overall, good agreement between both systems was achieved in our study, demonstrating the robustness and reliability of the proposed approach. Additionally, we demonstrated the invariance of the method by simulating rotations of the sensor on the foot. Taking advantage of this feature, and considering the wireless capabilities of the sensor, we postulate that the proposed solution is highly attractive for use at clinics and ambulatory settings. Its flexibility, combined with low errors achieved in the evaluation of gait parameters, may leverage trust and potentially simplify the data acquisition process. The solution should be evaluated with people with gait-related disorders, so that it may support clinical decision making in real scenarios.

## Figures and Tables

**Table 1:** Dataset characteristics after splitting.

Metric	Development Set (7 Subjects/45 Samples)	Validation Set (19 Subjects/115 Samples)
Height (cm)	168.9±7.7	172.5±9.2
Gender (female/male)	4/3	9/10
Weight (kg)	67.3±15.4	71.1±14.7
Foot size (cm)	27.4±1.6	27.8±2.0

**Table 2:** Methods and parameters used in coarse and fine grid-search.

Type	Methods	Coarse Grid-Search	Fine Grid-Search
Orientation estimation	Madgwick CF	${\bar{\omega }}_{max}=\left\{2,4,6\right\}$	${\bar{\omega }}_{max}=\left\{1.0,1.5,2.0,2.5,3.0\right\}$
	Euston CF	$k_{P}=\left\{0.2,0.4,0.6\right\}$ $k_{I}=\left\{0,0.05\right\}$	$k_{P}=\left\{0.15,0.2,0.25\right\}$ $k_{I}=\left\{0,0.01\right\}$
	Gyroscope integration	Not applicable	Not applicable
Double integration	Direct and reverse integration	η={0.1,0.2} $\alpha_{i}=\left\{0.4,0.5,0.6\right\}$	η={0.15,0.2,0.25} $\alpha_{i}=0.55,0.6,0.65$
	Linear dedrifting	Not applicable	Not applicable
	Horizontal correction	{True, False}	{True}

**Table 3:** Performance on the development set, using the best combination of methods and parameters (*n* = 2788 strides). Shown are mean values (standard deviation) and RMSE.

Metric	IMU	VICON	Rel. Error	Abs. Error	RMSE
Stride dur. (s)	1.23(0.27)	1.23(0.27)	0.00(0.03)	0.02(0.03)	0.03
Swing dur. (s)	0.41(0.08)	0.40(0.07)	0.01(0.04)	0.03(0.03)	0.04
Stance dur. (s)	0.82(0.21)	0.83(0.21)	−0.01(0.03)	0.02(0.02)	0.03
Cad. (st/min)	101.8(21.0)	101.7(20.9)	0.1(2.6)	1.7(1.9)	2.6
SL (cm)	112.4(25.2)	114.9(26.0)	−2.5(7.3)	5.3(5.6)	7.7
Speed (cm/s)	97.8(35.9)	100.2(37.9)	−2.3(6.9)	4.8(5.4)	7.3
SW (cm)	8.9(8.1)	9.2(8.5)	−0.3(1.3)	0.9(1.0)	1.3
MTC (cm)	1.7(0.8)	1.7(0.6)	0.1(0.8)	0.6(0.5)	0.8
Turn angle (${}^{\circ }$)	36.0(40.3)	35.3(40.3)	0.7(1.4)	1.0(1.2)	1.6

**Table 4:** Performance on the validation set, including turns (*n* = 7015 strides). Shown are mean values (standard deviation), limits of agreement, RMSE, correlation and equivalence interval (*p*-value). ^†^ All correlations were based on Spearman and have p<0.01. ^‡^ Equivalence tests were based on Wilcoxon signed-rank test.

Metric	IMU	VICON	Rel. Error	Abs. Error	Lim. Agr.	RMSE	Corr. ^†^	Equival. ^‡^
Stride dur. (s)	1.22(0.23)	1.23(0.24)	0.00(0.05)	0.02(0.05)	[−0.11,0.11]	0.05	0.99	±0.06(0.0)
Swing dur. (s)	0.41(0.08)	0.41(0.06)	0.01(0.05)	0.03(0.04)	[−0.08,0.09]	0.05	0.87	±0.02(0.0)
Stance dur. (s)	0.81(0.18)	0.82(0.19)	−0.01(0.05)	0.03(0.05)	[−0.11,0.10]	0.05	0.98	±0.04(0.0)
Cad. (st/min)	101.2(17.5)	101.1(17.4)	0.1(3.1)	1.8(2.5)	[−6.0,6.1]	3.1	0.99	±5.05(0.0)
SL (cm)	120.8(25.1)	124.2(26.5)	−3.5(9.7)	6.4(8.0)	[−22.5,15.6]	10.3	0.94	±6.21(0.0)
Speed (cm/s)	103.6(33.9)	106.7(36.3)	−3.1(9.2)	5.8(7.8)	[−21.1,15.0]	9.7	0.98	±5.33(0.0)
SW (cm)	9.9(9.2)	10.2(10.1)	−0.4(4.4)	1.5(4.2)	[−9.0,8.3]	4.4	0.93	±0.51(0.0)
MTC (cm)	1.7(1.0)	1.9(0.7)	−0.2(0.8)	0.6(0.6)	[−1.9,1.4]	0.9	0.55	±0.10(1.0)
Turn angle (${}^{\circ }$)	37.3(41.0)	36.4(41.0)	0.9(8.6)	2.3(8.4)	[−16.0,17.8]	8.7	0.97	±1.82(0.0)

**Table 5:** Performance on the validation set, excluding turning strides (*n* = 3785 strides). Shown are mean values (standard deviation), limits of agreement, RMSE, correlation and equivalence interval (*p*-value). ^†^ All correlations were based on Spearman and have p<0.01. ^‡^ Equivalence tests were based on Wilcoxon signed-rank test.

Metric	IMU	VICON	Rel. Error	Abs. Error	Lim. Agr.	RMSE	Corr. ^†^	Equival. ^‡^
Stride dur. (s)	1.22(0.24)	1.22(0.24)	0.00(0.04)	0.02(0.04)	[−0.08,0.08]	0.04	0.99	±0.06(0.0)
Swing dur. (s)	0.41(0.07)	0.40(0.06)	0.00(0.04)	0.02(0.04)	[−0.08,0.09]	0.04	0.89	±0.02(0.0)
Stance dur. (s)	0.81(0.19)	0.82(0.19)	0.00(0.04)	0.02(0.03)	[−0.09,0.08]	0.04	0.98	±0.04(0.0)
Cad. (st/min)	101.8(17.6)	101.5(17.4)	0.3(2.5)	1.5(2.1)	[−4.7,5.2]	2.5	0.99	±5.08(0.0)
SL (cm)	129.8(18.3)	133.7(20.7)	−3.9(6.2)	4.6(5.8)	[−16.1,8.2]	7.4	0.98	±6.69(0.0)
Speed (cm/s)	112.0(31.9)	115.3(35.1)	−3.4(6.9)	4.4(6.3)	[−17.0,10.2]	7.7	0.99	±5.77(0.0)
SW (cm)	4.9(3.2)	4.7(2.2)	0.1(2.8)	0.9(2.7)	[−5.4,5.6]	2.8	0.84	±0.24(0.0)
MTC (cm)	1.4(0.7)	1.8(0.6)	−0.4(0.7)	0.6(0.5)	[−1.7,0.9]	0.8	0.55	±0.09(1.0)
Turn angle (${}^{\circ }$)	6.4(7.0)	5.1(4.8)	1.2(5.1)	1.5(5.1)	[−8.8,11.3]	5.3	0.68	±0.26(1.0)

**Table 6:** Orientation invariance results (*n* = 7138 strides). Shown are mean values (standard deviation), RMSD, Correlation—with Spearman or Pearson—and equivalence interval (*p*-value). ^†^ All correlations have *p* < 0.01. ^‡^ All equivalence tests are based on Paired *T*-test, except for turning angle, which is based on Wilcoxon signed-rank test.

Metric	Original Orientation	Random Rotations	RMSD	Correlation ^†^	Equivalence ^‡^
Stride dur. (s)	1.23(0.26)	1.23(0.26)	0.0	$r_{p}=1.0$	±0.01(0.0)
Swing dur. (s)	0.41(0.08)	0.41(0.08)	0.0	$r_{p}=1.0$	±0.00(0.0)
Stance dur. (s)	0.82(0.20)	0.82(0.20)	0.0	$r_{p}=1.0$	±0.01(0.0)
Cad. (st/min)	100.7(17.9)	100.7(17.9)	0.0	$r_{p}=1.0$	±1.01(0.0)
SL (cm)	120.4(25.5)	120.4(25.5)	0.0	$r_{p}=1.0$	±1.20(0.0)
Speed (cm/s)	102.9(34.3)	102.9(34.3)	0.0	$r_{p}=1.0$	±1.03(0.0)
SW (cm)	9.8(9.2)	9.8(9.2)	0.0	$r_{p}=1.0$	±0.10(0.0)
MTC (cm)	1.7(1.0)	1.7(1.0)	0.0	$r_{p}=1.0$	±0.02(0.0)
Turn angle (${}^{\circ }$)	37.4(41.0)	37.4(41.0)	1.5	$r_{s}=0.99$	±0.37(0.0)

## Data Availability

Not applicable.

## Abbreviations

The following abbreviations are used in this manuscript:

CF	Complementary filter
FC	Initial foot contact
FO	Foot off
IMU	Inertial measurement unit
MTC	Minimum toe clearance
PCA	Principal component analysis
RMSD	Root mean square deviation
RMSE	Root mean square error
SL	Stride length
SW	Swing width
TRIAD	Tri-axial attitude determination
ZVI	Zero velocity interval
